# Influencing factors for pediatric eye disorders and health related quality of life: a cross-sectional study in Shanghai, China

**DOI:** 10.3389/fmed.2024.1420848

**Published:** 2024-07-30

**Authors:** Qin Shu, Zhongzhou Xiao, Xinwei Peng, Xiaoyi Liang, Moxin Chen, Zhuoran Tao, Qianwen Liu, Yonglin Guo, Xuefeng Yang, Wanqin Nie, Ruiyao Chen, Liya Yang, Jin Li, Jie Xu, Lin Li

**Affiliations:** ^1^Department of Ophthalmology, Shanghai Ninth People’s Hospital, Shanghai Jiao Tong University School of Medicine, Shanghai, China; ^2^Shanghai Key Laboratory of Orbital Diseases and Ocular Oncology, Shanghai, China; ^3^Shanghai Artificial Intelligence Laboratory, Shanghai, China

**Keywords:** pediatric eye diseases, myopia, strabismus, ptosis, risk factors, health-related quality of life (HRQoL), PedsQL 4.0

## Abstract

**Background:**

Myopia, strabismus, and ptosis are common pediatric eye diseases, which have a negative impact on children and adolescents in terms of visual function, mental health, and health-related quality of life (HRQoL). Therefore, this study focused on those pediatric eye diseases by analyzing their risk factors and HRQoL for the comprehensive management of myopia, strabismus, and ptosis.

**Methods:**

A total of 363 participants (2–18 years old) were included in this study for risk factors analysis of myopia, strabismus, and ptosis. We collected demographic characteristics, lifestyle habits and eye care habits of these children and analyzed them by using univariable and multivariable logistic regression. In addition, we applied the Chinese version of Pediatric Quality of Life Inventory-Version 4.0 (PedsQL 4.0) to assess HRQoL in 256 children with strabismus and ptosis. Univariable and multivariable linear regression models were applied to evaluate potential influencing factors of HRQoL.

**Results:**

Of all the participants, 140 had myopia, 127 had strabismus, and 145 had ptosis. Based on the multivariable logistic regression analysis model, we found that the history of parental myopia and daily average near-distance eye usage time were risk factors for myopia, and increased body mass index (BMI) was identified as a risk factor for strabismus and ptosis. Individuals with ptosis possessed decreased HRQoL. The multivariable linear regression model suggested that daily average near-distance eye usage time, light intensity during visual tasks, and daily average sleep duration had potential influences on HRQoL.

**Conclusion:**

This is the first study to assess the risk factors and HRQoL of myopia, strabismus, and ptosis together. We identified risk factors for these common pediatric eye diseases to help doctors, parents, and teachers better manage them. Our study discovered that children with eye disorders exhibit a notably diminished HRQoL. Consequently, it emphasizes the necessity for increased social attention and mental health assistance for these children.

## 1 Introduction

The pediatric eye health is a public concern worldwide. Myopia, strabismus, and ptosis are common eye diseases in children and adolescents. Specifically, myopia has a prevalence of over 50% among Chinese children and adolescents (1), and the prevalence of strabismus in children ranges from 1.19 to 5.65% (2–6), while ptosis affects around 0.18–1.41% of children (7–9).

Myopia, especially high myopia, frequently leads to various complications such as myopic macular degeneration, retinal tears, and retinal detachment, which can result in a deterioration or loss of vision (1). However, strabismus and ptosis frequently impair the typical development of children’s visual system, leading to compromised visual function (10, 11). Furthermore, the presence of strabismus and ptosis can significantly impact a child’s physical appearance, perhaps contributing to their psychological distress and increasing the likelihood of developing various mental health issues (12, 13). These disorders can significantly impair children’s overall growth and development, as well as their quality of life. Hence, when addressing pediatric eye problems, it is imperative to focus on two essential elements: identifying the variables that increase the likelihood of these diseases and understanding their influence on the growth and development of children.

The risk factors associated with these three prevalent pediatric eye diseases are multifaceted and diverse. Recent research has discovered various risk factors associated with myopia in children, such as family history of myopia, outdoor activity time (14), ethnicity (15), living in urban regions (16), socioeconomic status (17), and body stature (18, 19). Research has shown that gender, body mass index (BMI), and living in urban areas are factors related to the prevalence of ptosis (20). Strabismus is associated with a family history of strabismus (21), myopia (21), hyperopia (21), astigmatism (21, 22), amblyopia (22), families with lower parental education (22), prematurity, and smoking during pregnancy (21, 23). Presently, the majority of research is centered around the analysis of singular eye disorders, with just a limited number of studies examining a combination of numerous prevalent pediatric eye diseases. Nevertheless, we discovered that some children suffered from the combination of various eye disorders. Hence, it is imperative to conduct a comprehensive analysis of the risk factors associated with various eye diseases simultaneously. This will enhance our comprehension of the interactions and shared impacting variables among different ocular diseases enabling us to more effectively and thoroughly address the eye health of children and adolescents.

Other from the risk factors associated with pediatric eye diseases, the effect on the quality of life of children is a significant concern. Health-related quality of life (HRQoL) specifically refers to the aspect of quality of life that is related to one’s health by assessing the aspects of physical, mental, and social well-being. It has been reported that eye diseases have a negative influence on the HRQoL (24–26). Nevertheless, there was a shortage of studies regarding the HRQoL in children with pediatric eye diseases. Several studies have specifically examined the older adult population and have found that individuals with visual impairment frequently report a lower HRQoL (27, 28). However, due to variations in the way people of different ages perceive their quality of life, we believe that the research findings from adults cannot be immediately applied to the younger generation.

Therefore, the primary objective of this study was to identify the risk factors associated with myopia, strabismus, and ptosis in children and adolescents. Specifically, we focused on exploring the relationships between environmental factors, genetic factors, and eye care habits in relation to these three eye diseases. In the meantime, this study also aimed to evaluate the association between these eye diseases and the HRQoL, and explore their potential influencing factors. Our research can provide doctors with a more thorough understanding of how these diseases impact the entire health and well-being of children, and facilitate early detection and intervention, as well as create a supportive environment for affected children. Ultimately, this will lead to improved outcomes and quality of life for these children.

## 2 Methods

### 2.1 Study design and participants

From October 1, 2022 to September 30, 2023, we carried out a study using questionnaires at the Ophthalmology department of Shanghai Ninth People’s Hospital, School of Medicine, Shanghai Jiao Tong University. We developed the survey using the Delphi method as a guideline and modified it based on a review of existing literature. The survey was consisted of three main sections: demographic information, questions pertaining to pediatric eye diseases, and an inventory to assess HRQoL.

An anonymous online survey was conducted using WenJuanXing,^1^ an online platform in China for collecting data through questionnaires. The participants comprehended the questions effectively and provided accurate responses. Children over the age of 9 answered the questions themselves. However, children under the age of 9 answered the questions with the assistance of their parents. Typically, parents questioned children under 9 years old and filled out the questionnaire based on the children’s verbal responses to ensure the data’s authenticity. Furthermore, the responses were documented for the withdrawal from answering midway owing to various factors, which partially influenced the management of non-response and recall bias. The response and completion rates for all administered questionnaires were exceptionally high.

**Figure 1:**
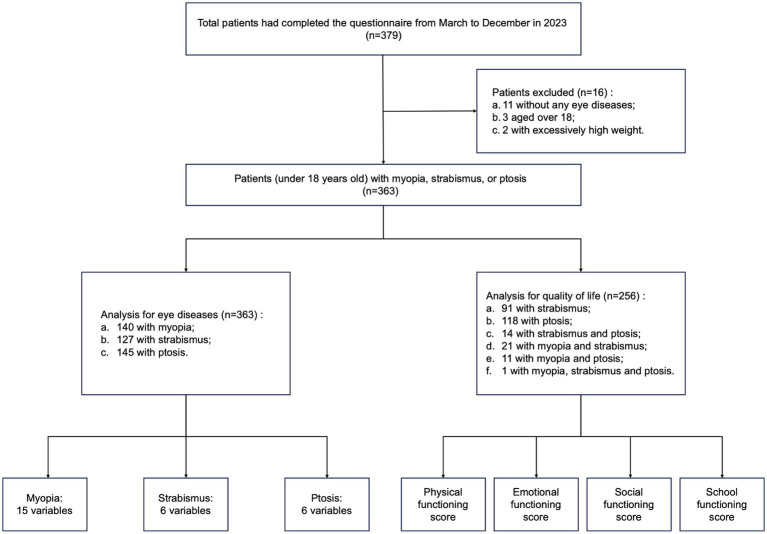
Study design.

Included patients were required to meet the four criteria: (1) age ≤ 18 years old; (2) had been diagnosed with myopia, strabismus, or ptosis at Shanghai Ninth People’s Hospital; (3) availability of complete medical records; and (4) informed consent obtained from parents or guardians and participants. This study eliminated parents or guardians who were hesitant to share the questionnaire data. Out of the 379 individuals who filled out the questionnaire, 363 of them satisfied the requirements and were included in the study. Regarding data pertaining to HRQoL, a total of 256 individuals met the criteria for eligibility (Figure 1).

This cross-sectional study received approval from the Ethics Committee of Shanghai Ninth People’s Hospital, School of Medicine, Shanghai Jiao Tong University (SH9H-2023-T99-1), and followed the principles outlined in the Declaration of Helsinki. This study adheres to the Strengthening the Reporting of Observational Studies in Epidemiology (STROBE) criteria and the Checklist for Reporting Results of Internet E-surveys (CHERRIES).

### 2.2 Measurements of pediatric eye diseases characteristics

The primary result focused on pediatric eye diseases. Within the questionnaire, we inquired, “Which of the subsequent pediatric eye diseases do you experience?” The options for this multiple-choice question (allowing for more than one correct response) were myopia, strabismus, ptosis, and none. Follow-up questions were dependent on the answer to this initial question, and any submissions without an answer will be excluded.

Demographic characteristics such as age, gender, height, and weight were collected from all participants. Additionally, information regarding eye use and lifestyle habits was gathered, including whether picky about food (Yes or No), whether supplements taken (Yes or No), sweets taken frequency, deep-fried food taken frequency, history of parental myopia, daily average near-distance eye usage time, daily average usage time of electronic devices, main electronic devices used, light intensity during visual tasks, daily average sleep duration, daily average outdoor time, and whether lying down while reading books/electronic screens. Furthermore, the questionnaire for children with strabismus included inquiries regarding the following factors: whether the participant was premature baby (Yes or No), whether the mother of the participant was with an advanced maternal age (Yes or No), and whether the mother smoked during pregnancy. We included inquiries on the history of parental ptosis, the presence of headaches (Yes or No), and whether raising head when watching blackboard or TV.

### 2.3 Health-related quality of life data collection

The secondary outcome was strabismus and ptosis patients’ HRQoL, which was measured by the Chinese version of Pediatric Quality of Life Inventory-Version 4.0 (PedsQL4.0). The reliability and validity of the Chinese version of the PedsQL4.0 have been verified, and the instrument has been used frequently in the scientific research of Chinese populations (29, 30). It involves 23 items that yield a total score and four separate modules scores for physical functioning (eight items), emotional functioning (five times), social functioning (five items), and school functioning (five items). The items were subjected to a linear transformation, resulting in a five-point rating scale ranging from 0 to 100. Specifically, the values were assigned as follows: 0 represents a situation where the problem almost always occurs, 25 indicates that the problem often occurs, 50 suggests that the problem sometimes occurs, 75 implies that the problem seldom occurs, and 100 signifies that the problem never occurs. Higher values indicate superior HRQoL.

### 2.4 Statistical analysis

The participant’s demographic characteristics and their replies to questions relating to eyes were summarized based on three primary eye diseases. Descriptive statistics were used to analyze the data. Frequency was reported for qualitative variables, while mean and standard deviations (SDs) were reported for normally distributed continuous data. For data that were not normally distributed, medians and interquartile ranges (IQRs) were reported instead.

Both univariable and multivariable logistic regressions were conducted to examine the relationship between the outcome (myopia, strabismus, and ptosis) and putative risk factors while controlling for confounders such as age, gender, BMI, and other relevant covariates. Likewise, risk factors that might be associated with the HRQoL total score were found using univariable and multivariable linear regression models. The final model included all variables that were found to be statistically significant in the univariable analysis. Using multivariable linear models, mean estimates were also calculated for each of the subscale scores with the factors of interest being various combinations of eye diseases. Furthermore, a subgroup analysis was conducted based on gender.

All statistical analyses were implemented with the uses of SAS, version 9.4 (SAS Institute) and R, version 4.2.1. A two-sided *p* < 0.05 was considered statistically significant.

## 3 Results

### 3.1 General characteristics of the study population

**Figure 2:**
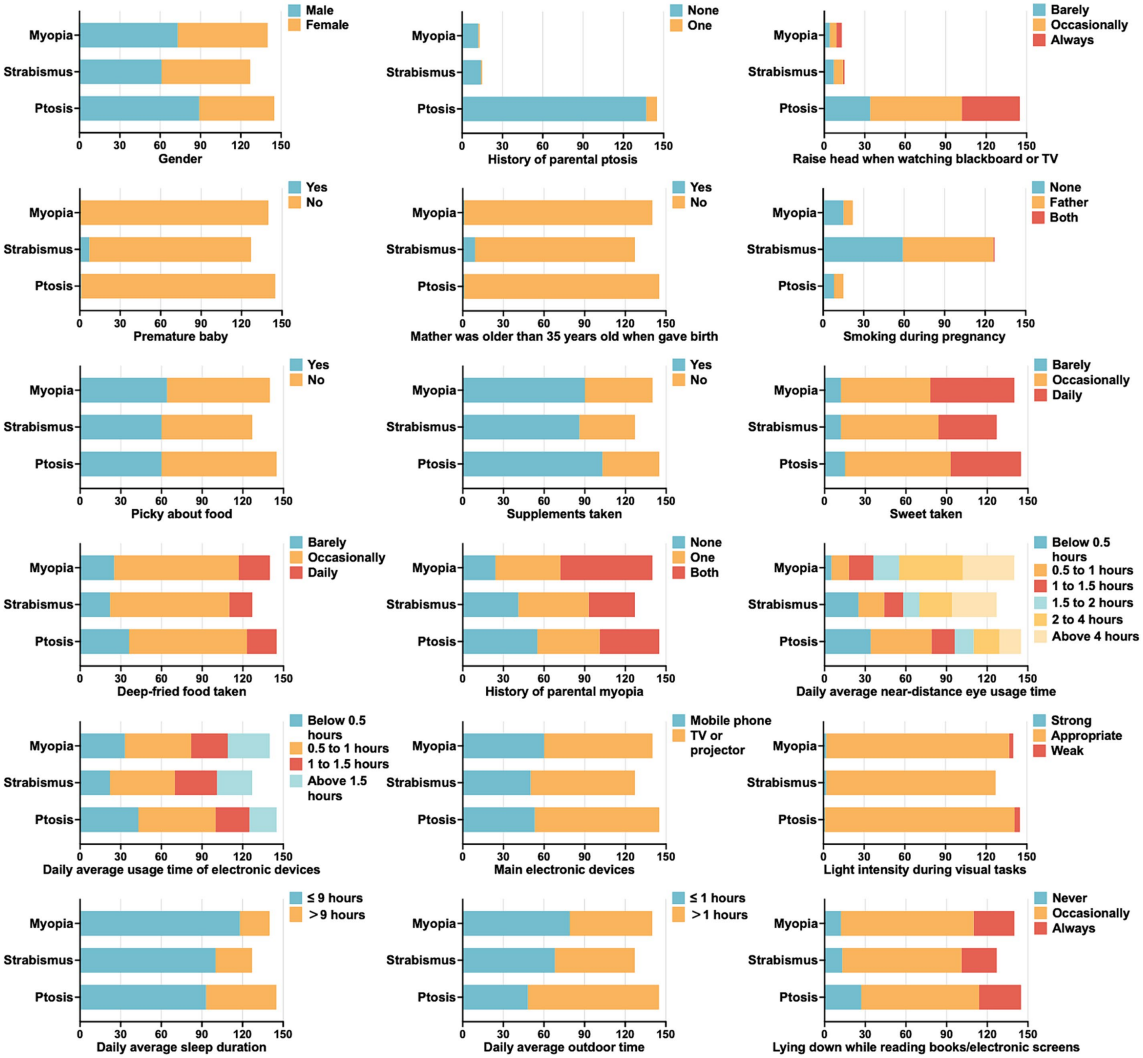
Eighteen characteristics of the participants with myopia, strabismus, and ptosis.

Table 1 and Figure 2 showed the characteristics of the study population with pediatric eye diseases. The mean (SD) age was 8.6 (3.6) years. Of these children, 168 (46.3%) were girls, while 195 (53.7%) were boys. A total of 154 (42.4%) children exhibited picky habits with food, whereas 247 (68.0%) children reported taking nutritional supplements. The majority of these children occasionally took sweets (194; 53.4%) or deep-fried food (233; 64.2%). In terms of the history of parental myopia, parents of 126 (34.7%) children were both myopic, and 127 (35.0%) children had one myopia parent, and the remaining 110 (30.3%) children had no myopic parent. A large proportion of individuals spent more than 4 h of daily average near-distance eye usage time (77; 21.2%), while 141 (38.8%) of the participants spent 0.5–1 h on electronic devices. Concerning the electric devices, 38.0% (138) children mainly used mobile phones, while 62.0% (225) used either a TV or projector. A majority of children (273; 75.2%) had an average of less than 9 h of sleep per day, and a great number of children (243; 66.9%) occasionally lied down while reading books or using electronic screens.

**Table 1:** Characteristics of the study participants.

Variables	Total	Myopia	Strabismus	Ptosis
	(*N* = 363)	(*N* = 140)	(*N* = 127)	(*N* = 145)
Age	8.6 (3.6)	10.1 (2.8)	8.8 (3.6)	7.1 (3.6)
Gender				
Female	168 (46.3)	67 (47.9)	66 (52.0)	56 (38.6)
Male	195 (53.7)	73 (52.1)	61 (48.0)	89 (61.4)
BMI	16.8 (15.3–19.2)	17.7 (15.4–20.3)	16.7 (15.5–19.4)	16.6 (15.1–18.9)
History of parental ptosis				
None	137 (37.7)	12 (92.3)	14 (93.3)	137 (94.5)
One	8 (2.2)	1 (7.7)	1 (6.7)	8 (5.5)
Headache (Yes)	8 (2.2)	1 (7.7)	2 (13.3)	8 (5.5)
Raise head when watching blackboard or TV				
Barely	34 (9.4)	4 (30.8)	7 (46.7)	34 (23.5)
Occasionally	68 (18.7)	5 (38.5)	7 (46.7)	68 (46.9)
Always	43 (11.9)	4 (30.8)	1 (6.7)	43 (29.7)
Premature baby (Yes)	7 (5.5)	0 (0)	7 (5.5)	1 (6.7)
Advanced maternal age (Yes)	9 (7.1)	1 (4.6)	9 (7.1)	1 (6.7)
Smoking during pregnancy				
None	59 (16.3)	15 (68.2)	59 (46.5)	8 (53.3)
Father	67 (18.5)	7 (31.8)	67 (52.8)	7 (46.7)
Both	1 (0.3)	0 (0)	1 (0.8)	0 (0)
Picky about food (Yes)	154 (42.4)	64 (45.7)	60 (47.2)	60 (41.4)
Supplements taken (Yes)	247 (68.0)	90 (64.3)	86 (67.7)	103 (71.0)
Sweets taken				
Barely	34 (9.4)	12 (8.6)	12 (9.4)	15 (10.3)
Occasionally	194 (53.4)	66 (47.1)	72 (56.7)	78 (53.8)
Daily	135 (37.2)	62 (44.3)	43 (33.9)	52 (35.9)
Deep-fried food taken				
Barely	73 (20.1)	25 (17.9)	22 (17.3)	36 (24.8)
Occasionally	233 (64.2)	92 (65.7)	88 (69.3)	87 (60.0)
Daily	57 (15.7)	23 (16.4)	17 (13.4)	22 (15.2)
History of parental myopia				
None	110 (30.3)	24 (17.1)	41 (32.3)	55 (37.9)
One	127 (35.0)	48 (34.3)	52 (40.9)	46 (31.7)
Both	126 (34.7)	68 (48.6)	34 (26.8)	44 (30.3)
Daily average near-distance eye usage time				
Below 0.5 h	57 (15.7)	5 (3.6)	25 (19.7)	34 (23.4)
0.5–1 h	71 (19.6)	13 (9.3)	19 (15.0)	45 (31.0)
1–1.5 h	44 (12.1)	18 (12.9)	14 (11.0)	17 (11.7)
1.5–2 h	39 (10.7)	19 (13.6)	12 (9.4)	14 (9.7)
2–4 h	75 (20.7)	47 (33.6)	24 (18.9)	19 (13.1)
Above 4 h	77 (21.2)	38 (27.1)	33 (26.0)	16 (11.0)
Daily average usage time of electronic devices				
Below 0.5 h	85 (23.4)	33 (23.6)	22 (17.3)	43 (29.7)
0.5–1 h	141 (38.8)	49 (35.0)	48 (37.8)	57 (39.3)
1–1.5 h	75 (20.7)	27 (19.3)	31 (24.4)	25 (17.2)
Above 1.5 h	62 (17.1)	31 (22.1)	26 (20.5)	20 (13.8)
Main electronic devices				
Mobile phone	138 (38.0)	60 (42.9)	50 (39.4)	53 (36.6)
TV or projector	225 (62.0)	80 (57.1)	77 (60.6)	92 (63.5)
Light intensity during visual tasks				
Strong	4 (1.1)	2 (1.4)	2 (1.6)	1 (0.7)
Appropriate	354 (97.5)	135 (96.4)	125 (98.4)	140 (96.6)
Weak	5 (1.4)	3 (2.1)	0 (0)	4 (2.8)
Daily average sleep duration				
≤9 h	273 (75.2)	118 (84.3)	100 (78.7)	93 (64.1)
>9 h	90 (24.8)	22 (15.7)	27 (21.3)	52 (35.9)
Daily average outdoor time				
≤1 h	174 (47.9)	79 (56.4)	68 (53.5)	48 (33.1)
>1 h	189 (52.1)	61 (43.6)	59 (46.5)	97 (66.9)
Lying down while reading books/electronic screens				
Never	47 (12.9)	12 (8.6)	13 (10.2)	27 (18.6)
Occasionally	243 (66.9)	98 (70.0)	88 (69.3)	87 (60.0)
Always	73 (20.1)	30 (21.4)	26 (20.5)	31 (21.4)

BMI, Body mass index.

The number of children affected by myopia, strabismus, and ptosis was 140, 127, and 145, respectively. Compared to all participants, those who diagnosed with myopia were generally older (with an average age of 10.1 years old), had a higher BMI of 17.7 kg/m^2^, spent more than 2 h of daily average near-distance eye usage time (60.7 vs. 41.9%), spent less than 1 h outdoor every day (56.4 vs. 47.9%), and slept for less than 9 h (84.3 vs. 72.5%). In addition, the percentage of having a family history of myopia among children with myopia was significantly greater compared to the overall group (82.9 vs. 69.7%).

### 3.2 Association between pediatric eye diseases and risk factors

#### 3.2.1 Association between myopia and risk factors

**Figure 3:**
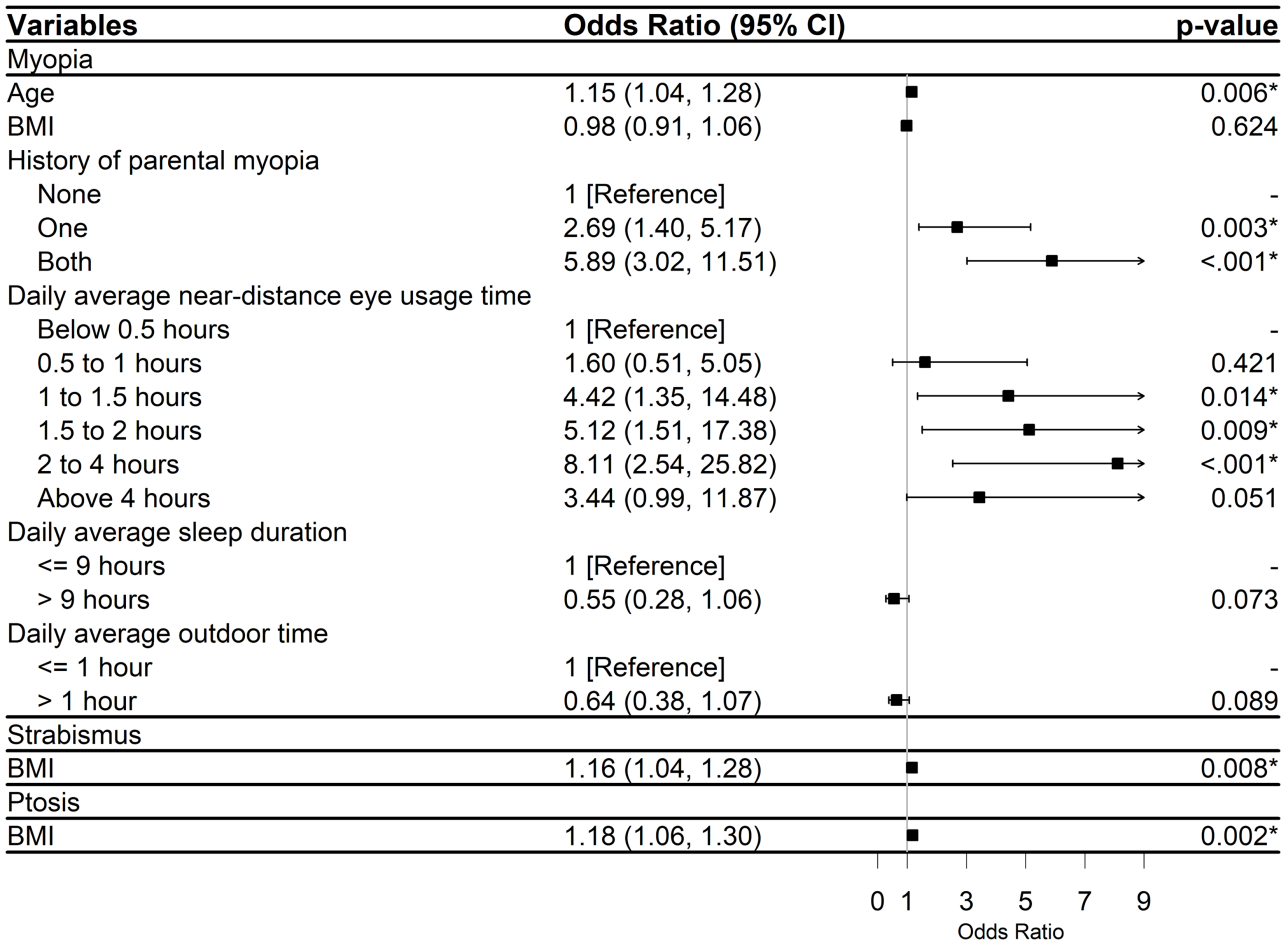
Multivariable logistic regression results for three pediatric eye diseases. CI, Confidence interval; BMI, Body mass index. ^*^p < 0.05.

After conducting an initial analysis using univariable logistic regression (Supplementary Figure 1A), we selected six variables (age, BMI, history of parental myopia, daily average near-distance eye usage time, daily average sleep duration, and daily average outdoor time) to be included in the final multivariable model for studying the risk factors of myopia (Figure 3). As a result, we found the older age was correlated with an increased risk of myopia (OR, 1.15; 95% confidence interval [95% CI], 1.04–1.28; *p* = 0.006). Meanwhile, a notable influence of genetic factors was suggested. Compared with children with none myopic parents, children with one myopic parent were 2.69 times more likely to develop myopia (OR, 2.69; 95% CI, 1.40–5.17; *p* = 0.003), and children with two myopic parents were 5.89 times more likely to develop myopia (OR, 5.89; 95% CI, 3.02–11.51; *p* < 0.001). In addition, limited daily average near-distance eye usage time were associated with decreased myopia risks.

**Figure 4:**
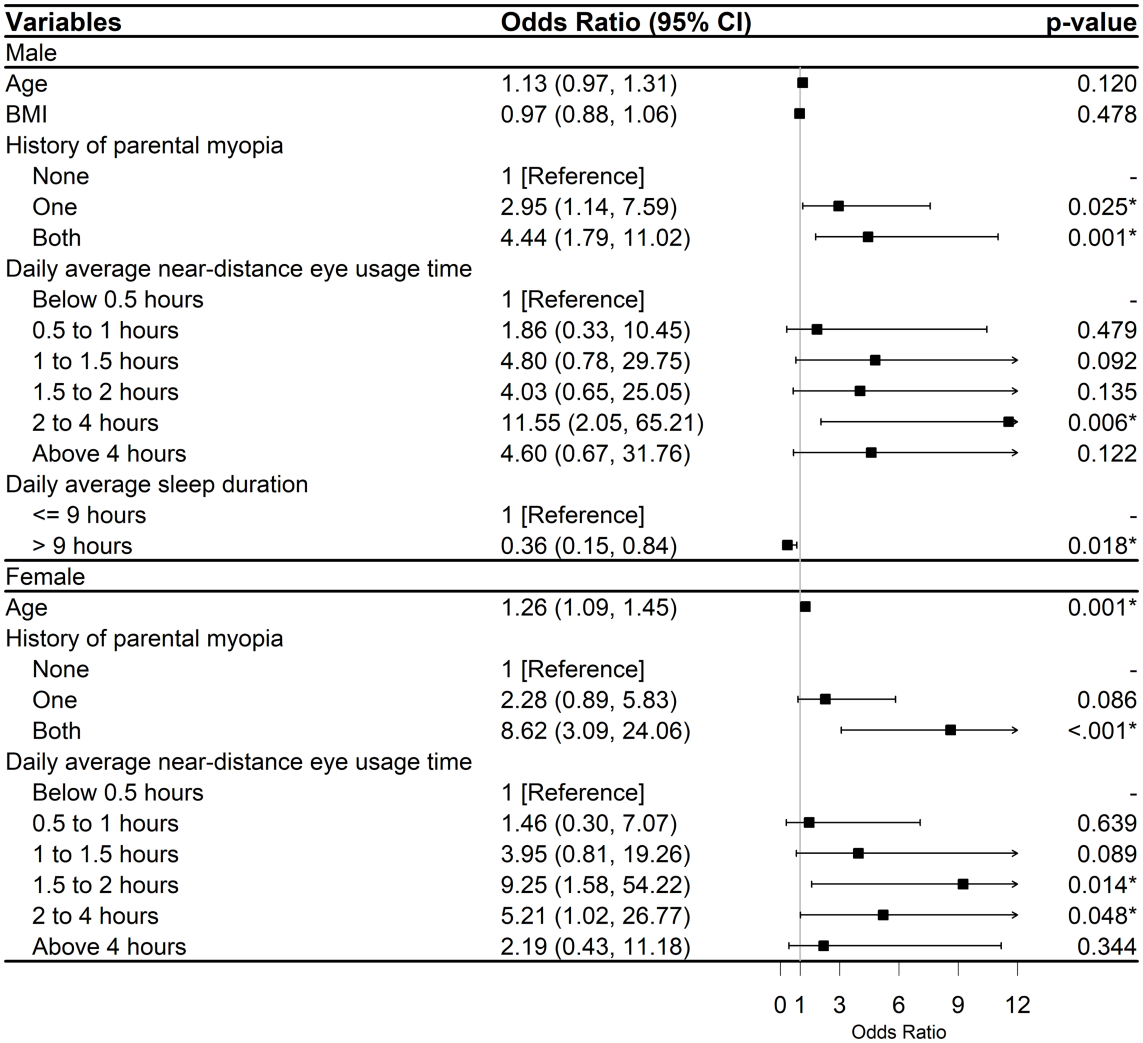
Analysis of gender subgroups among individuals with myopia. CI, Confidence interval; BMI, Body mass index. ^*^p < 0.05.

The subgroup analysis results, which were divided by gender, indicated that the factors affecting the occurrence of myopia were not identical for males and females (Figure 4). The findings indicated a positive correlation between age and the likelihood of myopia in females (OR, 1.26; 95% CI, 1.09–1.45; *p* = 0.001). Male individuals who slept more than 9 h per day had a lower likelihood of developing myopia (OR, 0.36, 95% CI: 0.15–0.84; *p* = 0.018), while this association was not observed in females. Nevertheless, in both males and females, history of parental myopia and increased daily average near-distance eye usage time were risk factors for myopia.

#### 3.2.2 Association between strabismus and risk factors

Supplementary Figure 1B summarized the findings of univariable logistic regression analysis and showed two variables (BMI and smoking during pregnancy) that associated with the primary outcome. In the multivariable logistic regression analysis (Figure 3), BMI was defined as the only one important factor (OR, 1.16; 95% CI, 1.04–1.28; *p* = 0.008).

#### 3.2.3 Association between ptosis and risk factors

For the analysis of influencing factors related to the occurrence of ptosis, both univariable (Supplementary Figure 1C) and multivariable (Figure 3) logistic regression analyses suggested that BMI was a significant influencing factor (OR, 1.18; 95% CI, 1.06–1.30; *p* = 0.002). As BMI increased, the likelihood of ptosis increased.

### 3.3 Health-related quality of life

**Figure 5:**
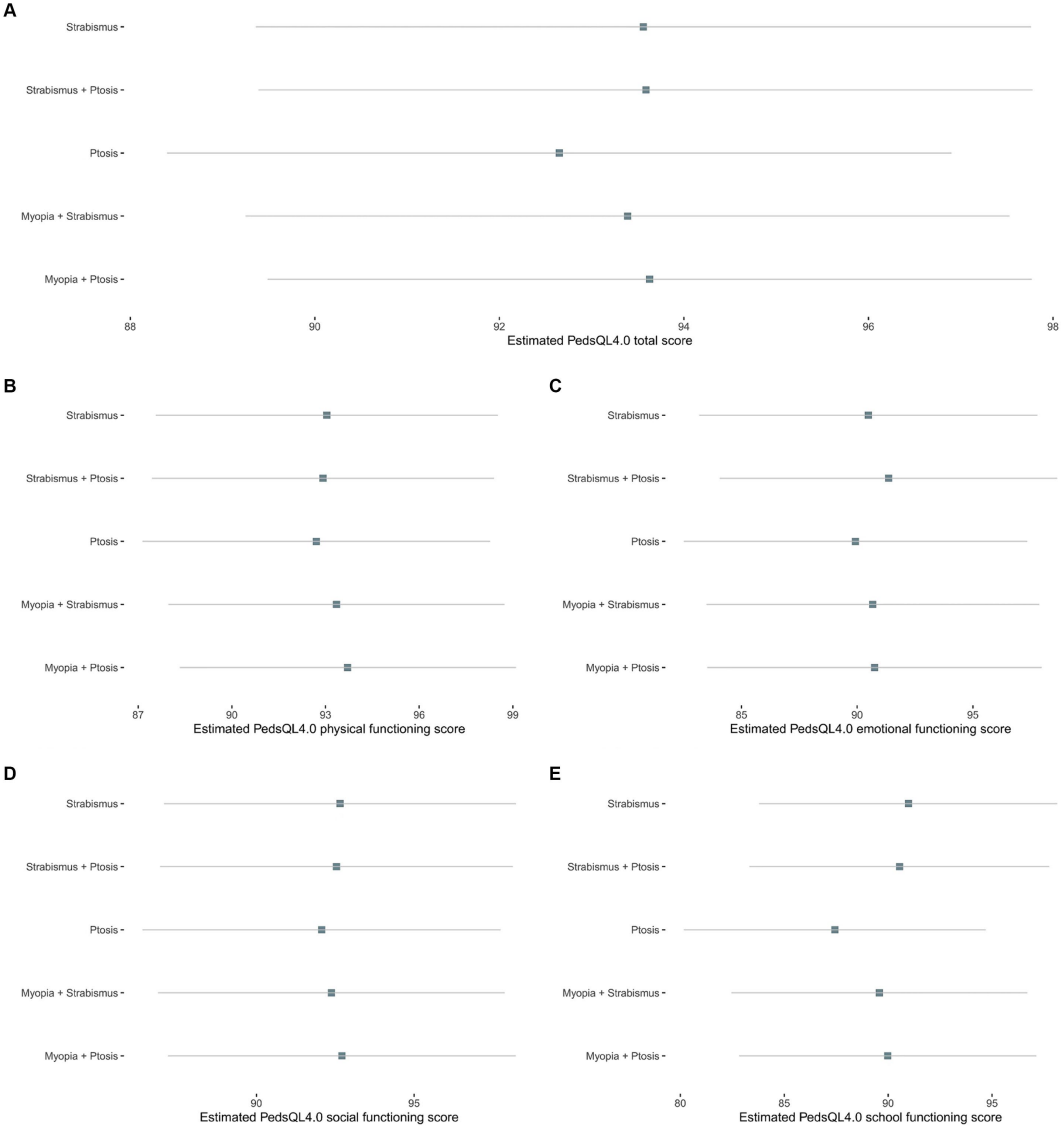
Estimated Pediatric Quality of Life–Version 4.0 (PedsQL-4.0) scores. **(A)** Total score; **(B)** Physical functioning; **(C)** Emotional functioning; **(D)** Social functioning; and **(E)** School functioning.

Data of HRQoL were collected based on 256 children, including 91 with strabismus, 118 with ptosis, 14 with strabismus and ptosis, 21 with myopia and strabismus, 11 with myopia and ptosis, and 1 with myopia, strabismus and ptosis (Figure 1). The children with ptosis reported the lowest PedsQL 4.0 score in the total and four separate modules, especially in the school functioning part (87.43; 95% CI, 80.17–94.70) (Figure 5; Supplementary Table 1). Other groups attained similar scores across modules, with slightly lower in the emotional and school functioning subscales.

**Figure 6:**
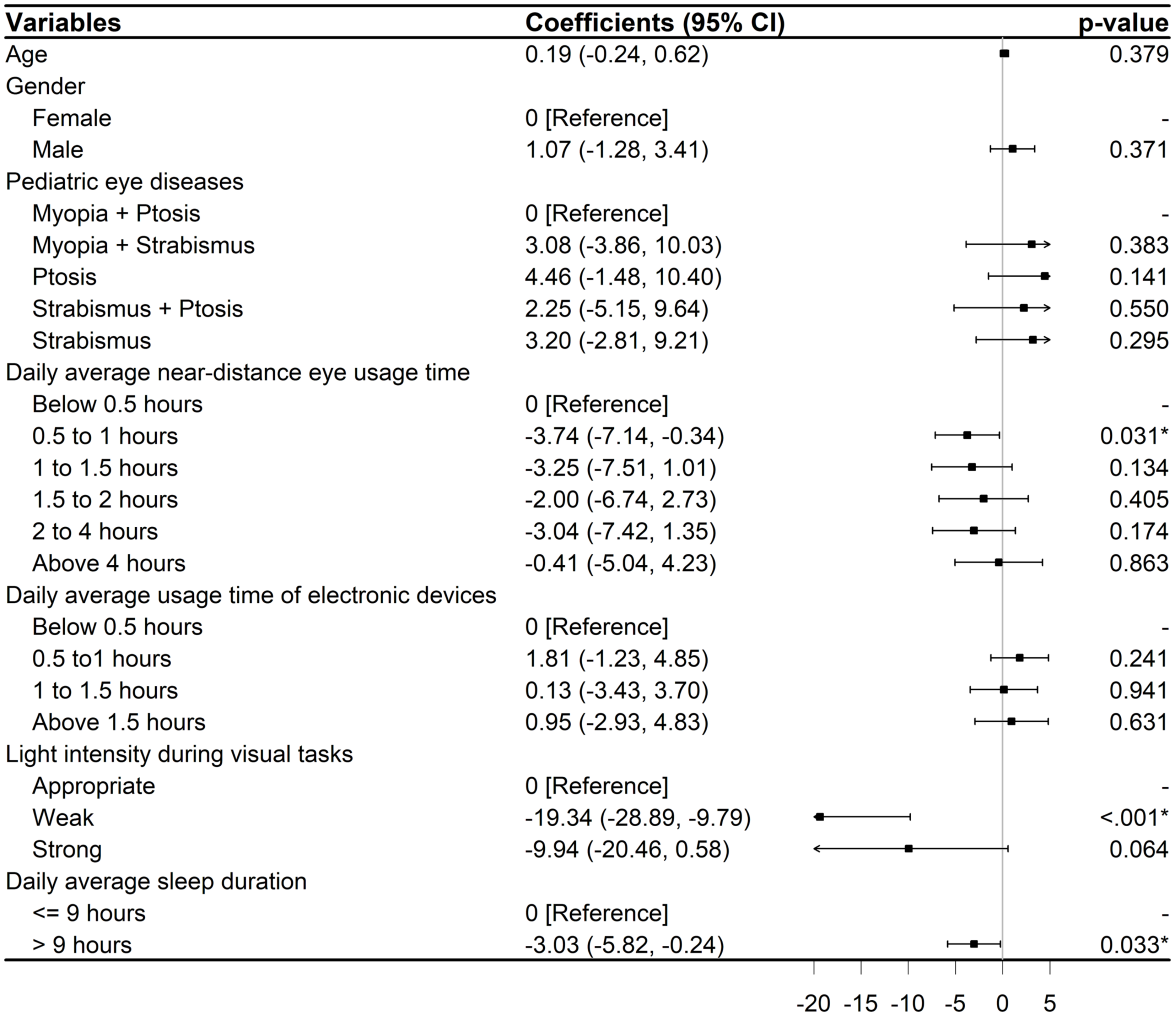
Multivariable linear regression results for HRQoL. CI, Confidence interval. ^*^p < 0.05.

The univariable linear regression model revealed significant correlations between children’s HRQoL and pediatric eye diseases, daily average near-distance eye usage time, light intensity during visual tasks, and daily average sleep duration (Supplementary Figure 2). The final multivariable model showed that children’s HRQoL was linked to an average of 0.5–1 h of near-distance eye usage (−3.74; 95% CI, −7.14 to −0.34; *p* = 0.031), and sleeping for more than 9 h on average (−3.03; 95% CI, −5.82 to −0.24; *p* = 0.033). In addition, weak light intensity during visual tasks significantly reduced the PedsQL 4.0 total score by 19.34 points (95% CI, −28.89 to −9.79; *p* < 0.001). No correlation was detected between any of the other variables (Figure 6).

## 4 Discussion

To the best of our knowledge, this is the first study to examine the HRQoL of children with strabismus and ptosis using PedsQL 4.0 and to simultaneously evaluate risk factors for myopia, strabismus, and ptosis. Our study revealed that history of parental myopia and increased daily average near-distance eye usage time were risk factors for the development of myopia. Furthermore, increased BMI was the potential risk factor for the occurrence of strabismus and ptosis. In addition, we found that children with ptosis had the lowest HRQoL. The daily average near-distance eye usage time and light intensity during visual tasks were significant contributors to HRQoL. The aforementioned findings assist doctors in gaining a deeper comprehension of the risk factors associated with common pediatric eye diseases. This knowledge enables them to categorize and manage different populations, as well as educate children and parents about proper eye habits. Meanwhile, the results of this study emphasize the need for increased focus on the HRQoL of children with strabismus and ptosis, as well as the provision of psychological support.

### 4.1 Pediatric eye diseases and risk factors

Myopia development is influenced by both hereditary and environmental factors. Prior research has demonstrated that children who have one or both parents with myopia have a higher likelihood of having myopia, compared to children whose parents do not have myopia (31–33). An analysis of a diverse population, encompassing various racial backgrounds such as Asian, African American, and Hispanic, revealed that children with a single myopic parent had a 0.42 times higher likelihood of developing early-onset myopia, while those with two myopic parents had a 1.70 times higher likelihood (32). Parental myopia was also found to be a risk factor for myopia in children and adolescents in our study. Our analysis suggested that this phenomenon may be related to genetic inheritance. However, it may also be related to environmental factors such as the generally higher educational level of myopic parents and more stressful home study habits. In addition, the relationship between myopia and near eye usage was controversial. Huang et al. (34) proposed that near-eye usage had a minimal impact on the progression of myopia, while Ip et al. (35) argued that there was no correlation between myopia and the overall duration of near-eye usage. Our study showed that decreasing the daily average near-distance eye usage time lowered the risk of myopia.

Within the gender subgroup analysis, daily average sleep duration and age were significant influences on the occurrence of myopia in males and females, respectively. Regarding sleep, a 2-year prospective trial in China showed that staying up late was a risk factor for myopia, but total sleep duration had no significant effect (36). A Korean study showed that a decrease in total sleep duration increased the likelihood of myopia (37).

Our study indicated that having a higher BMI was associated with a greater probability of developing strabismus and ptosis. A study in China demonstrated that obesity was a significant risk factor for the occurrence of concomitant exotropia (38).

Furthermore, earlier research has established a correlation between increased BMI and the occurrence of ptosis in adults (39–41). However, there is currently a lack of relevant research among children and adolescents.

### 4.2 Health-related quality of life of affected children

Our research revealed that children with ptosis had the lowest HRQoL, especially in terms of school functioning. Children with ptosis experience impaired visual field as a result of their eyelids obstructing a portion of their vision (42). This might potentially hinder the development of their eyesight, which in turn may impact their ability to attend school. What is more, because ptosis is so obvious in appearance (43), it may make socializing difficult for affected children. All of these reasons are significant in reducing the HRQoL of children with ptosis. In addition, previous studies have reported the application of PedsQL in the assessment of HRQoL in children with strabismus and found that children with strabismus had a reduced HRQoL (44, 45). Our analysis confirmed this observation.

In this investigation, we found that insufficient light intensity during visual tasks was a significant factor linked with a reduction in HRQoL in affected children. This finding motivated us to develop tailored interventions to improve HRQoL of children by improving light intensity during eye use.

### 4.3 Strengths and limitations

Our study has several strengths. First, previous work has predominantly examined the factors influencing a single eye disease, whereas our study conducted a complete analysis on three common pediatric eye diseases (myopia, strabismus, and ptosis). Second, this study revealed that an increased BMI is associated with a higher risk of ptosis and strabismus in children. Third, this study was the initial one to utilize PedsQL 4.0 for evaluating the quality of life in children with ptosis. Nevertheless, there were certain constraints. The vast majority of participants in this study were from Shanghai, China, which is a highly developed region in China. However, both the geography and socioeconomic position can influence pediatric eye disease or HRQoL. In the future, we will collect questionnaire data from other provinces and cities in China to conduct a more comprehensive multicenter study. In addition, we could not draw a causal relationship due to the cross-sectional study design.

## 5 Conclusion

To summarize, our study identified risk factors for common pediatric eye diseases, including myopia, strabismus and ptosis. Additionally, we examined the impact of these conditions on the HRQoL of affected children and studied the factors that influence their well-being. History of parental myopia and increasing daily average near-distance eye usage time were shown to be risk factors for the development of myopia. Elevated BMI was found to be a notable contributing factor in children with strabismus and ptosis. Moreover, children with ptosis had a notably decreased HRQoL. Hence, we urge public health policymakers, physicians, educators, and parents to effectively address these risk factors and offer enhanced psychological assistance to affected children.

## Data availability statement

The datasets discussed in this article are not readily available because they contain sensitive information that may violate the privacy of the individuals involved in the research. Please refer all requests to access the datasets to LL at lin_li@sjtu.edu.cn.

## Ethics statement

The studies involving humans were approved by the Ethics Committee of Shanghai Ninth People’s Hospital, School of Medicine, Shanghai Jiao Tong University (SH9H2023-T99-1). The studies were conducted in accordance with the local legislation and institutional requirements. Written informed consent for participation in this study was provided by the participants’ legal guardians/next of kin.

## Author contributions

QS: Conceptualization, Data curation, Investigation, Methodology, Writing – original draft. ZX: Data curation, Methodology, Visualization, Writing – original draft. XP: Data curation, Methodology, Visualization, Writing – original draft. XL: Data curation, Investigation, Writing – review & editing. MC: Data curation, Investigation, Writing – review & editing. ZT: Data curation, Investigation, Writing – review & editing. QL: Data curation, Investigation, Writing – review & editing. YG: Data curation, Investigation, Writing – review & editing. XY: Data curation, Investigation, Writing – review & editing. WN: Data curation, Investigation, Writing – review & editing. RC: Formal analysis, Software, Writing – review & editing. LY: Formal analysis, Software, Writing – review & editing. JL: Conceptualization, Project administration, Resources, Supervision, Validation, Writing – review & editing. JX: Conceptualization, Project administration, Resources, Supervision, Validation, Writing – review & editing. LL: Conceptualization, Funding acquisition, Project administration, Resources, Supervision, Validation, Writing – review & editing.

## Footnotes

^1^ https://www.wjx.cn

## Supplementary material

SUPPLEMENTARY FIGURE 1 Univariable logistic regression results for three pediatric eye diseases. CI, Confidence interval; BMI, Body mass index. ^*^*p* < 0.05.

SUPPLEMENTARY FIGURE 2 Univariable linear regression results for HRQoL. CI, Confidence interval; BMI, Body mass index. ^*^*p* < 0.05.
