# Supplementary material for: Influencing factors for pediatric eye disorders and health related quality of life: a cross-sectional study in Shanghai, China
Source: Front Med (Lausanne). 2024 Jul 30;11:1420848. doi: 10.3389/fmed.2024.1420848 (PMC11319247; doi:10.3389/fmed.2024.1420848)

Supplementary Table 1 Total and Subscale Estimated Mean on the PedsQL 4.0^a^ at different pediatric eye diseases groups

| **Groups** | **Mean Estimated (95% CI)** |
| --- | --- |
| **PedsQL 4.0 Total Score** |  |
| Strabismus | 93.56 (89.36 to 97.76) |
| Strabismus + Ptosis | 93.59 (89.39 to 97.78) |
| Ptosis | 92.65 (88.40 to 96.90) |
| Myopia + Strabismus | 93.39 (89.25 to 97.53) |
| Myopia + Ptosis | 93.63 (89.49 to 97.77) |
| **PedsQL 4.0 Physical Functioning Score** |  |
| Strabismus | 93.04 (87.56 to 98.52) |
| Strabismus + Ptosis | 92.92 (87.44 to 98.40) |
| Ptosis | 92.70 (87.14 to 98.27) |
| Myopia + Strabismus | 93.35 (87.97 to 98.73) |
| Myopia + Ptosis | 93.71 (88.33 to 99.10) |
| **PedsQL 4.0 Emotional Functioning Score** |  |
| Strabismus | 90.48 (83.17 to 97.79) |
| Strabismus + Ptosis | 91.36 (82.51 to 97.35) |
| Ptosis | 89.93 (82.51 to 97.35) |
| Myopia + Strabismus | 90.67 (83.48 to 97.87) |
| Myopia + Ptosis | 90.75 (83.52 to 97.98) |
| **PedsQL 4.0 Social Functioning Score** |  |
| Strabismus | 92.65 (87.07 to 98.23) |
| Strabismus + Ptosis | 92.54 (86.95 to 98.13) |
| Ptosis | 92.07 (86.39 to 97.74) |
| Myopia + Strabismus | 92.38 (86.88 to 97.87) |
| Myopia + Ptosis | 92.71 (87.20 to 98.22) |
| **PedsQL 4.0 School Functioning Score** |  |
| Strabismus | 90.97 (83.79 to 98.14) |
| Strabismus + Ptosis | 90.55 (83.33 to 97.76) |
| Ptosis | 87.43 (80.17 to 94.70) |
| Myopia + Strabismus | 89.58 (82.46 to 96.70) |
| Myopia + Ptosis | 89.98 (82.83 to 97.13) |

**Supplementary Figure 1**


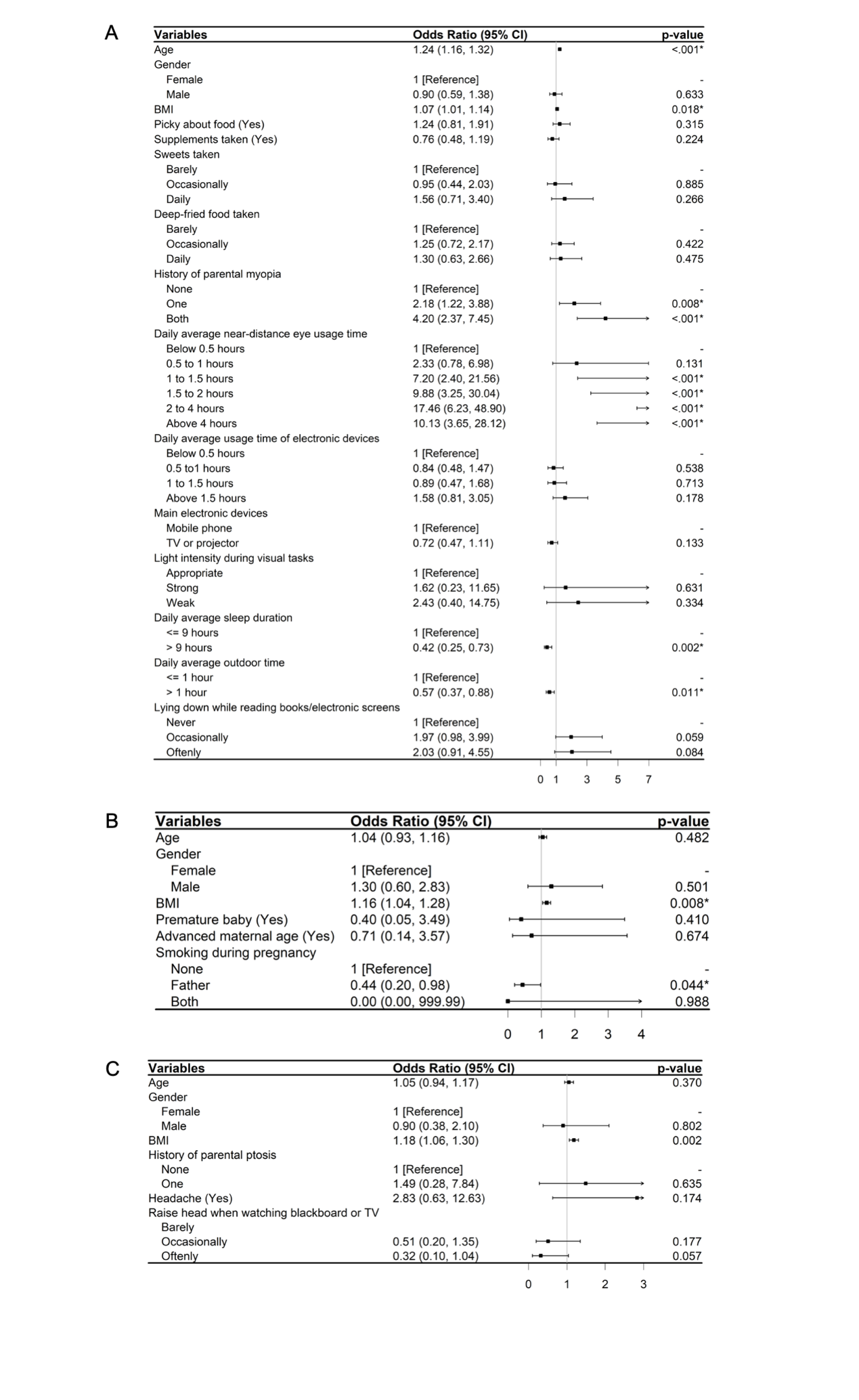


**Supplementary Figure 2**


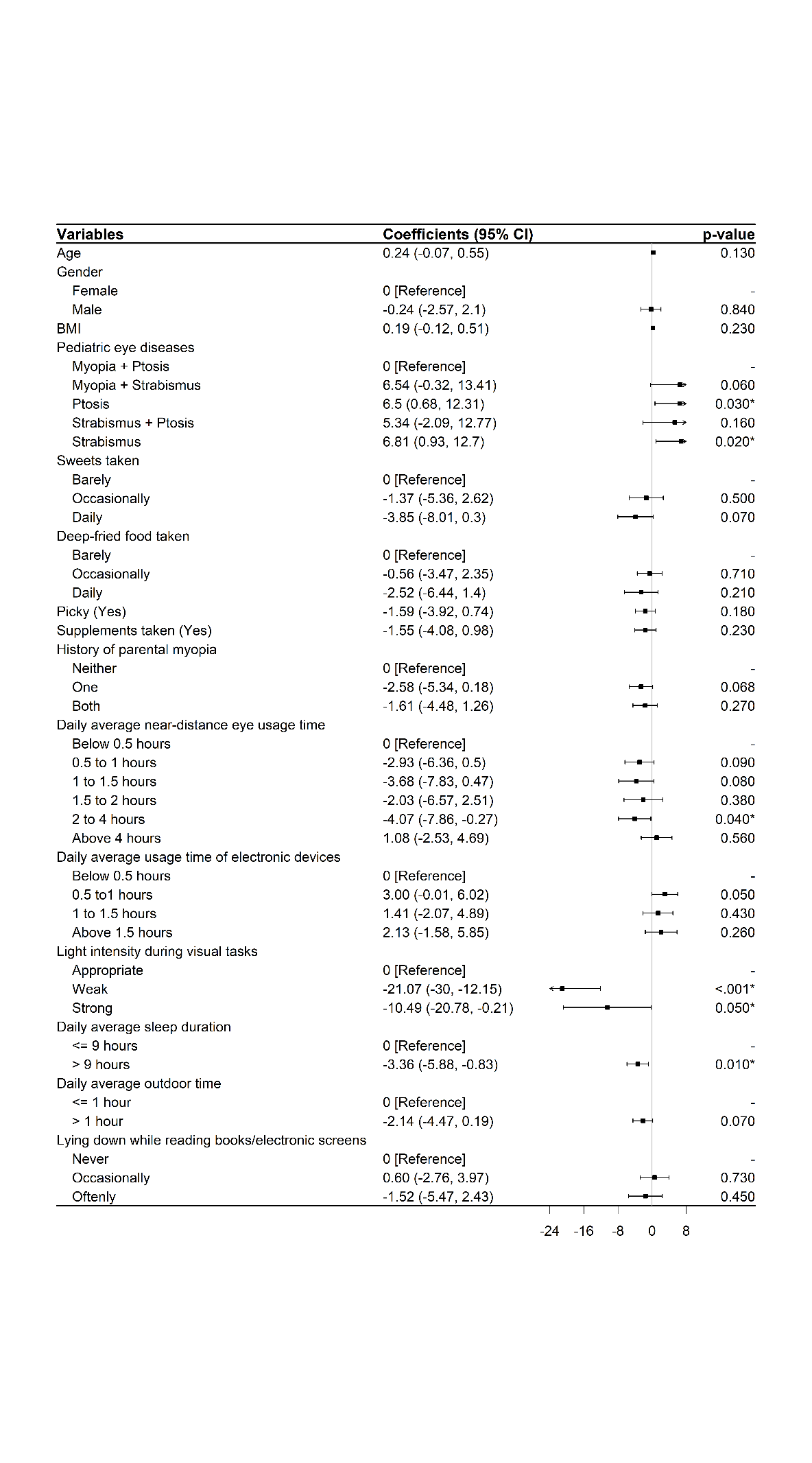

Supplement: SUPPLEMENTARY FIGURE 1 — Univariable logistic regression results for three pediatric eye diseases. CI, Confidence interval; BMI, Body mass index. *p < 0.05. [file Data_Sheet_1.docx]
